# Supplementary material for: Time‑resolved multi-omic analysis of paclitaxel exposure in human iPSC‑derived sensory neurons unveils mechanisms of chemotherapy‑induced peripheral neuropathy
Source: Cell Death Dis. 2026 Feb 10;17(1):211. doi: 10.1038/s41419-026-08445-2 (PMC12921266; doi:10.1038/s41419-026-08445-2)
Supplement: Supplementary file 15 — Dataset 1. [file 41419_2026_8445_MOESM15_ESM.zip › CDD_Open_Data/05_Protein/03_Evidence_Plots_Andranik/GOBP_AXO_DENDRITIC_PROTEIN_TRANSPORT.pdf]

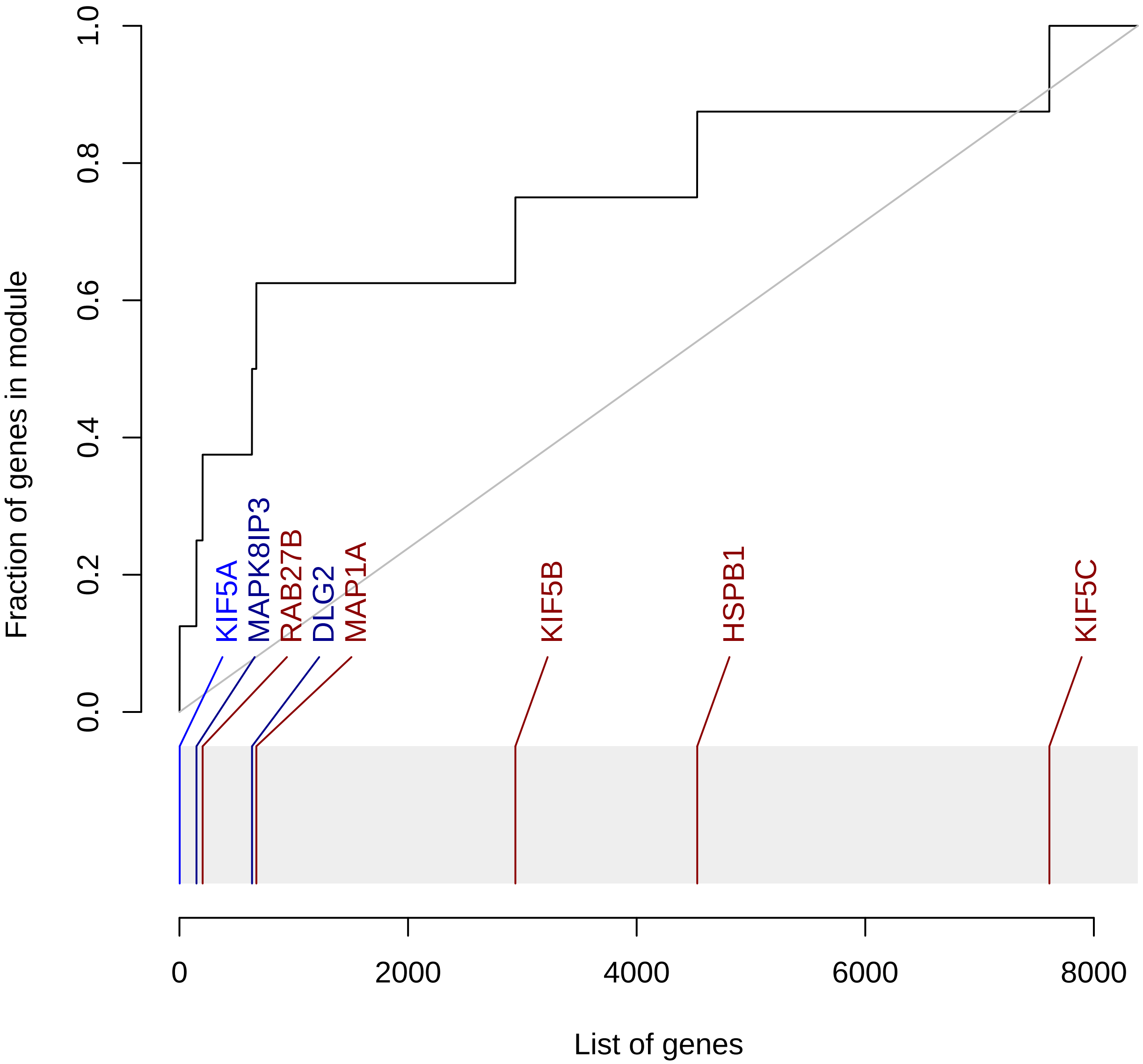

Fraction of genes in module
1.0
0.8
0.6
0.4
0.2
0.0
KIF5A
MAPK8IP3
RAB27B
DLG2
MAP1A
KIF5B
HSPB1
KIF5C
0
2000
4000
6000
8000
List of genes
